# Assessment of HER2/Neu Expression in Colorectal Carcinomas and Its Correlation With Tumor Stage and Histopathology

**DOI:** 10.7759/cureus.79721

**Published:** 2025-02-26

**Authors:** Lakshmi Sai Vijay Achalla, Raju K Shinde, Samarth Shukla, Sangita D Jogdand, Sahitya Vodithala

**Affiliations:** 1 Department of General Surgery, Jawaharlal Nehru Medical College, Datta Meghe Institute Of Higher Education and Research, Wardha, IND; 2 Department of Pathology, Jawaharlal Nehru Medical College, Datta Meghe Institute Of Higher Education and Research, Wardha, IND; 3 Department of Pharmacology, Jawaharlal Nehru Medical College, Datta Meghe Institute Of Higher Education and Research, Wardha, IND

**Keywords:** colorectal adenocarcinoma, her2/neu, immunohistochemistry, targeted therapy, trastuzumab

## Abstract

Introduction

Colorectal carcinoma (CRC) is the second leading cause of cancer-related mortality worldwide. Within this context, a subset of CRCs shows overexpression of the human epidermal growth factor receptor 2 (HER2/neu), a characteristic also seen in other malignancies like breast and gastric cancers. The success of targeting the HER2 pathway in these cancers has prompted investigations into the potential use of similar interventions in CRC.

Aims

This research aims to assess HER2/neu expression in CRCs and its correlation with the CRC stage and histopathology, as well as to evaluate the demographic characteristics of CRC patients.

Materials and methods

This prospective observational study was conducted on a cohort of 40 CRC patients, using histopathology and immunohistochemistry (IHC) sections from the Department of Pathology. Clinical and demographic data were collected over a 24-month period, and routine tissue processing and IHC staining were performed on colectomy tissues. Immunostaining with the HER2/neu marker was conducted for detailed analysis. Data were analyzed using IBM SPSS Statistics for Windows, Version 27.0 (Released 2020; IBM Corp., Armonk, NY, United States).

Results

Among the 40 CRC cases studied, six cases (15%) exhibited robust membranous positivity, while eight cases (20%) showed moderate focal membranous positivity. Twenty-six cases (65%), however, demonstrated no HER2/neu staining positivity. A significant correlation was found between the histological grade of the tumor and HER2/neu expression (p = 0.0001). Additionally, HER2/neu expression was significantly correlated with lymphovascular invasion (p < 0.0001) and lymph node status (p < 0.047).

Conclusion

This study found a strong correlation between the tumor's stage, grade, lymph node status, and lymphovascular invasion and HER2/neu expression. Therefore, HER2/neu can be a predictive and therapeutic marker in colorectal cancers. This underscores the potential importance of incorporating these parameters in the clinical evaluation and targeted treatment strategies for CRC patients.

## Introduction

Colorectal carcinoma (CRC) accounts for approximately 10% of global malignancies and 9.4% of all cancer-related deaths worldwide, second only to lung carcinoma, which contributes to 18% of cancer-related mortality [1]. In 2020, 1.93 million new CRC cases were diagnosed, resulting in 0.94 million deaths, with a projected increase to 3.08 million cases by 2040 [1].

CRC is more prevalent in nations with a higher Human Development Index (HDI) compared to those with a lower HDI. While CRC prevalence is declining in highly developed countries, it is rising in less developed regions, such as India. The most common histological type of CRC is adenocarcinoma, which is initiated by oncogenic events such as serrated adenomas, the adenoma-carcinoma sequence, and inflammation. Uncontrolled oncogenic events lead to the accumulation of genetic mutations and epigenetic modifications, transforming cells into uncontrollable adenomas and, ultimately, CRC [1].

Numerous risk factors for CRC have been identified, including age, low-fiber diet, inactivity, obesity, excessive alcohol consumption, and smoking. Regular physical activity has consistently been associated with a reduced risk of CRC, likely through mechanisms such as inhibiting fat accumulation, suppressing inflammation, enhancing bowel motility, and modulating metabolic hormones and insulin regulation. However, it is important to recognize that CRC risk reduction is influenced by a complex interplay of lifestyle, genetic, and environmental factors, with physical activity being one modifiable component of a comprehensive prevention strategy [2].

While risk factors such as lifestyle, genetic predisposition, and environmental exposures significantly contribute to the development of colorectal cancer, they also influence tumor biology and, consequently, treatment outcomes. For example, certain genetic alterations associated with CRC risk, such as RAS and BRAF mutations, are known to predict resistance to targeted therapies and impact overall survival. Similarly, lifestyle factors such as obesity and physical inactivity can modulate systemic inflammation and metabolic profiles, which may affect treatment efficacy and disease progression [2].

However, treatment outcomes and prognosis in CRC are largely dependent on the disease stage at diagnosis, emphasizing the importance of preventive strategies to reduce new cases [2]. 

Despite advancements in surgical and chemotherapeutic approaches, a significant proportion of cancer patients show poor response to treatment. Progress in tumor biology has led to the identification of novel therapeutic targets, emphasizing the critical need for molecular biomarkers that predict outcomes, therapeutic responses, and potential targets [3-5].

The human epidermal growth factor receptor 2 (HER2/neu) proto-oncogene, located on chromosome 17q21, codes for ErbB-2, a member of the EGFR family. HER2 activation plays a pivotal role in cell proliferation, differentiation, apoptosis inhibition, and tumor progression [6].

Trastuzumab, a humanized monoclonal antibody, targets the extracellular domain of the HER2 receptor. In breast carcinoma, HER2 gene amplification and protein overexpression, observed in 15%-20% of cases, correlate with an aggressive phenotype, metastasis, and poor outcomes. The successful application of Herceptin in breast and gastric cancers underscores the need to explore HER2/neu blockade as a potential clinical tool in gastrointestinal cancers [7,8].

Limited studies on HER2/neu overexpression in CRCs in the Indian subcontinent warrant further investigation [9]. HER2/neu overexpression is associated with increased cell survival and proliferation, as well as reduced apoptotic potential, contributing to neoplastic transformation. HER2/neu can manifest in both membranous and cytoplasmic forms, with distinct implications in various tumors. While cytoplasmic HER2/neu expression is common in breast cancer, it is often overlooked, as therapeutic monoclonal antibodies primarily target membranous forms. However, in colorectal adenocarcinoma (CRAC), HER2/neu expression occurs in both membranous and cytoplasmic forms. Despite this, HER2/neu testing in CRAC has not been extensively studied in the South Asia region, with very few studies published on HER2/neu overexpression in CRCs from this area [9]. 

This study aims to assess the frequency of HER2/neu overexpression in CRAC and explore its correlation with clinicopathological features in this demographic.

## Materials and methods

This prospective observational study was conducted in collaboration between the Department of General Surgery and the Department of Pathology at Jawaharlal Nehru Medical College, Datta Meghe Institute of Higher Research and Education (DMIHER), Sawangi (Meghe), Wardha, India. Approval was obtained from the Institutional Ethical Committee of DMIHER (IEC no. DMIMS(DU)/IEC/Sept-2019/8415). A cohort of 40 patients undergoing surgery for colorectal cancer over a period of two years (October 2019 to November 2021) was selected.

All resected specimens of CRC with a histopathological diagnosis of CRAC received at the Department of Pathology, Jawaharlal Nehru Medical College, Sawangi (Meghe), Wardha, during the study period were included.

Patients who underwent chemotherapy or radiotherapy before surgery were excluded, as these treatments can alter the tumor microenvironment, including HER2/neu expression. Such therapies may downregulate or upregulate HER2/neu levels, potentially leading to inaccurate assessment of its natural expression and its correlation with histopathological features. Patients who underwent colonoscopic biopsies were also excluded, as these yield small tissue samples that may not provide sufficient material for reliable HER2/neu testing or comprehensive histopathological evaluation.

**Figure 1:**
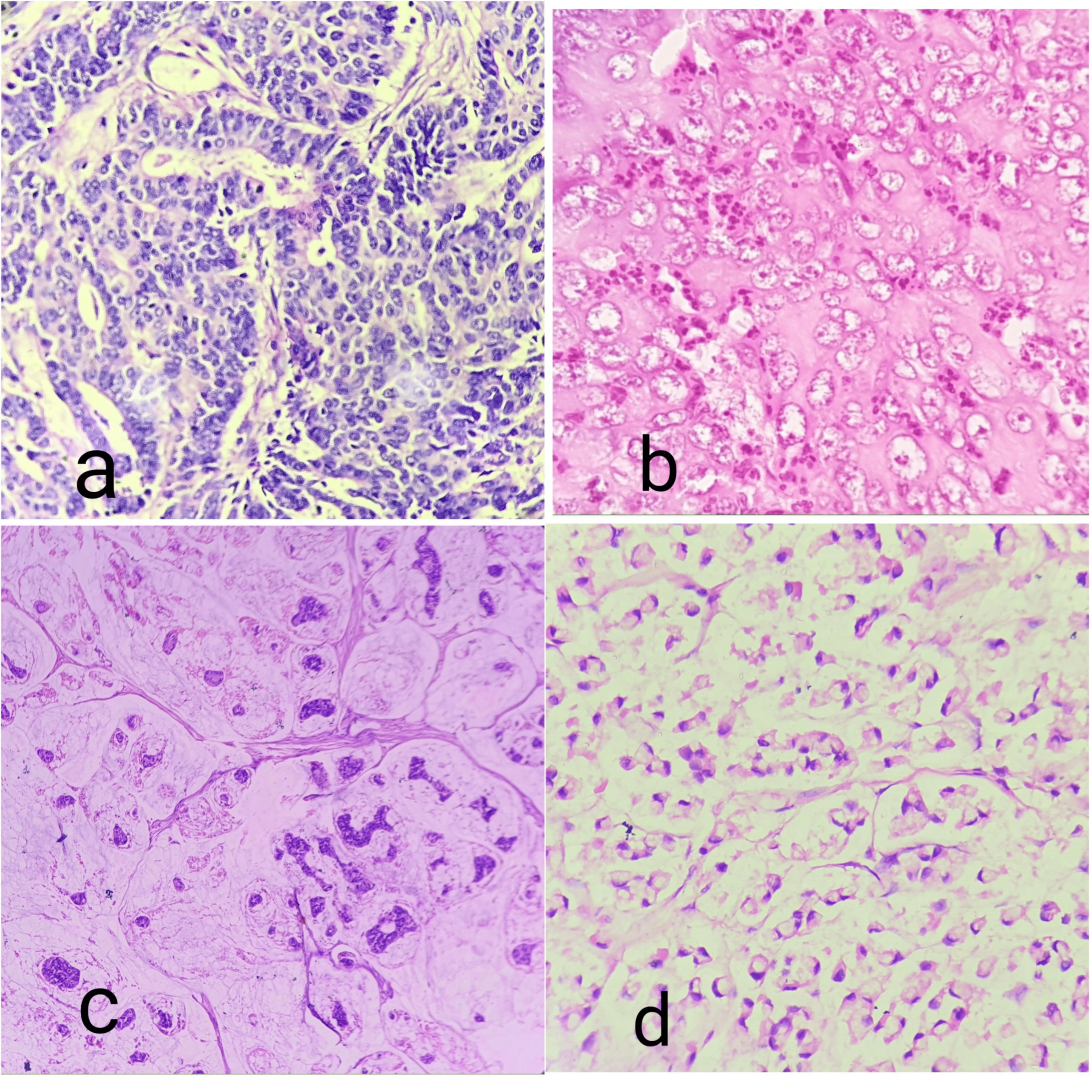
Pictomicrograph showing (a) moderately differentiated adenocarcinoma (40×), (b) poorly differentiated adenocarcinoma (40×), (c) histology of mucinous adenocarcinoma (40×), and (d) histology of signet ring cell adenocarcinoma (40×).

**Figure 2:**
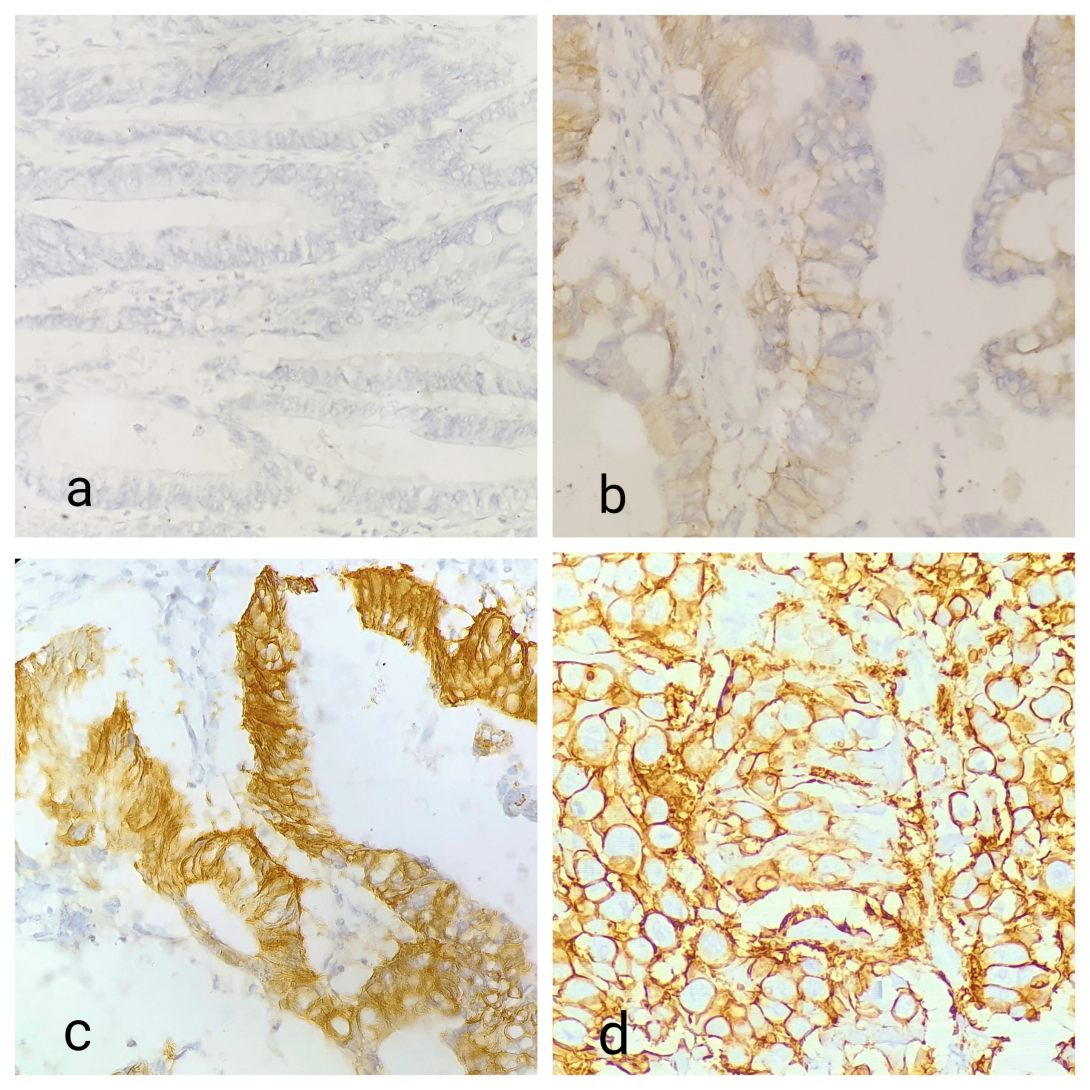
Pictomicrograph showing (a) HER2/neu IHC score 0 (40×), (b) HER2/neu IHC score 1+ (40×), (c) HER2/neu IHC score 2+ (40×), and (d) HER2/neu IHC score 3+ (40×). IHC: immunohistochemistry.

A total of 40 cases meeting the inclusion criteria were selected for further evaluation. Patient information, including age, gender, clinical findings, blood investigations, imaging, and colonoscopic findings, was systematically collected. After surgical resection, specimens were sent to the Pathology Department for fixation in 10% neutral buffered formalin for a minimum of 8-12 hours before grossing. The grossing of specimens and documentation of macroscopic features were meticulously performed. Tissue sections were extracted from selected areas of resected specimens containing tumors. Following routine tissue processing, 4-5 micron thick sections were cut from paraffin-embedded selected tumor blocks and stained with standard hematoxylin and eosin (H&E). Immunohistochemistry (IHC) staining for HER2/neu expression was performed on all CRAC cases using HER2/ErbB2-EP3 rabbit monoclonal antibody (PathnSitu Biotechnologies, Secunderabad, India) and an advanced polymer staining system (Figure 1 and Figure 2).

The guidelines for interpreting HER2 immunostaining were followed (Table 1). Immunostaining was observed in a homogeneous membranous population of cancer cells, and HER2 immunostaining was interpreted using a low-power objective (100×). IHC was employed to measure the expression of HER2/neu membrane proteins in cancer cells. The staining intensity and percentage were assessed using a scoring system from 0 to 3+. An expression score of 0 or 1+ for HER2/neu was considered negative, while a score of 3+ was considered positive. A score of 2+ was considered ambiguous and required confirmation using fluorescent in situ hybridization (FISH) or other readily available in situ hybridization methods. However, our experiment did not employ in situ hybridization.

**Table 1:** Immunohistochemical staining for HER2/neu expression: analysis and scoring. Source: [10].

Score	Staining pattern	Interpretation
0	No staining or membrane staining in <10% of tumor cells	Negative
1+	Faint staining or membrane staining in >10% of tumor cells	Negative
2+	Weak-to-moderate circumferential, lateral, or basolateral membranous staining in >10% of tumor cells	Equivocal
3+	Strong circumferential, lateral, or basolateral membranous staining in >10% of tumor cells	Positive

Statistical analysis

All collected data were properly maintained in a Microsoft Excel worksheet (Microsoft Corp., Redmond, WA, United States). Descriptive and inferential statistics were employed for data analysis. The chi-square test was used to assess the association between clinicopathological parameters. Statistical analyses were performed using IBM SPSS Statistics for Windows, Version 27.0 (Released 2020; IBM Corp., Armonk, NY, United States) and GraphPad Prism 7.0 version (Dotmatics, Boston, MA, United States). A p-value of <0.05 was considered statistically significant.

## Results

A total of 40 CRC cases were examined in this prospective study: 19 (47.5%) were male and 21 (52.5%) were female cases. The majority (67.5%) were over 50, with the mean age being 56.87 years (range 27-83 years). The most afflicted area was the sigmoid colon, which was seen in 11 out of 40 cases (27.5%), followed by the rectum (10%), the cecum (15%), the ascending colon (10%), the transverse colon (10%), the descending colon (10%), and the hepatic flexure of the colon (2.5%). Non-mucinous adenocarcinoma was the most prevalent histological subtype, occurring in 34 out of 40 cases (85%), followed by mucinous adenocarcinoma in six cases (15%). Twenty-six of the 40 cases (65%) had well-differentiated tumors.

Clinical presentations were characterized by prevalent symptoms, such as abdominal pain in 30 cases (75%), altered bowel habits in 29 cases (72.5%), and weight loss in 28 cases (70%). Carcinoembryonic antigen (CEA) marker levels were assessed in all 40 cases, revealing abnormal CEA levels in 30 cases, with the highest abnormal levels noted in the ascending colon (100%), followed by the sigmoid colon (90.91%). The highest mean CEA value was found in the cecum (35.63ng/mL), while the lowest was observed in the hepatic flexure.

On colonoscopy, the most common macroscopic finding was cauliflower growth (ulceroproliferative) in 47.5% of cases, followed by lumen narrowing growth in 30%. The stage-wise distribution of colorectal cancers in our study showed 50% of cases were stage III cancer, followed by 25% in stage II. Lymph node involvement was observed in 52.5% of tumors (21 out of 40 cases), and the lymph nodes were noted in 47.5% of tumors (19 out of 40 cases). Following HER2/neu IHC analysis, one case (2.5%) had a 3+ score, five cases (12.5%) had a 2+ score, eight cases (20%) had a 1+ score, and the remaining 26 cases (65%) had a score of 0 (Table 2).

**Table 2:** Distribution of patients based on HER2/neu status. This study found 2.5% of cases with strong membranous positivity (3+) and 12.5% with moderate membranous positivity, while the majority (65%) showed no positivity on HER2/neu staining.

HER2/neu status	No. of cases	Percentage
0	26	65
1+	8	20
2+	5	12.5
3+	1	2.5
Total	40	100

The relationship between HER2/neu expression and the CRC tumor site in the right-side colon was statistically significant (p = 0.032). Among right-sided colon patients, two had 2+ and one had 3+ HER2/neu positive, whereas five had no expression and five had a 1+ expression. The histological grade of the tumor was significantly correlated with HER2/neu expression (p = 0.0001). Poorly differentiated adenocarcinoma exhibited 3+ HER2/neu expression across various tumor histological grades. Additionally, a significant association was found with lymph node status (p = 0.0001), of which one case with 3+ HER2/neu status was classified as N2. A case with 3+ HER2/neu positivity was positive for lymphovascular invasion (p = 0.047), and a significant correlation was found between AJCC tumor stage and HER2/neu positivity (p = 0.008) (Table 3).

**Table 3:** Association of HER2/neu status with clinicopathological features. This table shows the association of HER2/neu expression with other clinicopathological features such as gender, age, CEA levels, histology, colonoscopic findings, histological grade, tumor site, tumor stage, lymph node status, lymphovascular invasion, and depth of tumor invasion with HER2/neu positivity. LVI: lymphovascular invasion, NS: not significant, S: significant, CEA: carcinoembryonic antigen.

Clinicopathological features	HER2/neu status				p-value
	0	1+	2+	3+	
Gender					
Male	11	4	3	1	0.63, NS
Female	15	4	2	0	
Age (years)					
≤60	16	5	3	1	0.89, NS
>60	10	3	2	0	
Histology					
Mucinous	4	2	0	0	0.63, NS
Non-mucinous	22	6	5	1	
CEA status					
Normal (0-3)	7	2	1	0	0.92, NS
Abnormal (>3)	19	6	4	1	
Tumor grade					
I	21	5	0	0	0.0001, S
II	5	3	5	0	
III	0	0	0	1	
Tumor site					
Colon	18	7	4	1	0.67, NS
Rectum	8	1	1	0	
Left-side colon	21	3	3	0	0.032, S
Right-side colon	5	5	2	1	
Depth of invasion					
T1	0	0	0	0	0.36, NS
T2	10	1	1	1	
T3	14	5	4	0	
T4	2	2	0	0	
T1-2	10	1	1	1	0.23, NS
T3-4	16	7	4	0	
Lymph node status					
N0	19	0	0	0	0.0001, S
N1	4	5	5	0	
N2	3	3	0	1	
Present	7	8	5	1	0.0001, S
Absent	19	0	0	0	
Tumor stage					
I	8	0	0	0	0.008, S
II	11	0	0	0	
III	7	7	5	1	
IV	0	1	0	0	
LVI					
Present	6	2	3	1	0.047, S
Absent	20	6	2	0	
Colonoscopy findings					
Cauliflower growth (Ulceroproliferative)	10	4	4	1	0.61, NS
Lumen narrowing growth	9	2	1	0	
Polypoidal growth	7	2	0	0	

The depth of invasion and HER-2/neu expression did not show a significant correlation (p = 0.36). Furthermore, no statistical relationships were found between colonoscopic findings, CEA status, age, gender, or the tumor's histological diagnosis.

## Discussion

CRC remains a prevalent malignancy despite advancements in diagnostic and therapeutic modalities. Carcinogenesis arises from the complex interplay of environmental and genetic factors. Extensive research has shed light on the fundamental aspects of tumor biology and pathogenesis in CRCs [11].

Histopathological diagnosis, while crucial, reveals that CRCs express diverse immunological markers, influencing therapeutic decisions and prognostication. Among these markers, HER2/neu emerges as a functional indicator, offering insights into the outcomes of colorectal tumors. In this study, the PathnSitu HER2/neu antibody for IHC staining was employed, utilizing specific criteria defined by Valtorta et al. [10], known as the HERACLES (HER2 Amplification for Colorectal cancer Enhanced Stratification) diagnostic criteria. Notably, in situ hybridization studies were not performed on the 2+ and 3+ HER2/neu stains.

The overexpression of HER2/neu indicates an unfavorable prognosis and helps identify patients who may benefit from immunotherapy using targeted monoclonal antibody therapy. A comparative analysis with other studies showed a 15% HER2/neu overexpression in CRC patients. Among this subset, 2.5% exhibited strong membranous positivity, and 12.5% displayed moderate membranous positivity for HER2/neu immunohistochemistry. This contrasts with the findings of Pappas et al. [12] and Schuell et al. [13], who recorded overexpression at around 4%. Our results are consistent with previous studies conducted by Li et al. [14] and Lazaris et al. [15], which reported HER2/neu overexpression in 15.5% and 11.6% in Tu et al. [16] (Table 4). 

**Table 4:** Comparative study of HER2/neu status overexpression with other studies.

S. no.	Studies	HER2/neu overexpression (%)
1.	Pappas et al. [12]	3.9
2.	Schuell et al. [13]	4
3.	Li et al. [14]	15.5
4.	Lazaris et al. [15]	15.5
5.	Tu et al. [16]	11.6
6.	Present study	15

Variations in tissue fixation intensity, antibodies, and IHC techniques, as well as staining patterns (cytoplasmic, membranous, or both), may all contribute to the broad range of HER2/neu overexpression. The study population's variability, sample size, racial disparities, and technical variations in IHC performance could all be responsible for these discrepancies [17,18].

In our study, moderate membranous staining (2+) was identified in cases of moderately and well-differentiated adenocarcinomas, while strong membranous staining (3+) was observed in one case of poorly differentiated adenocarcinoma. This research underscores the complex landscape of HER2/neu expression in CRCs, revealing varied patterns across histological differentiations. The integration of molecular markers like HER2/neu into clinical decision-making processes holds significant potential for developing individualized treatment strategies for CRC patients.

Comparative study of the association of HER2/neu status with clinicopathological features

In the present study, a comprehensive analysis was conducted to assess the correlation between HER2/neu status and various clinicopathological features. Notably, no significant associations were observed between HER2/neu positivity and age, gender, histology type (mucinous/non-mucinous), CEA levels, tumor site, colonoscopic findings, and depth of invasion (T stage). The statistical analysis yielded p-values of 0.89, 0.63, and 0.92 for age, gender, and histology, respectively, aligning with similar findings reported by Wang et al. (p-values of 0.27, 0.44, and 0.762, respectively) [17]. In contrast, Kountourakis et al. reported a statistically significant expression of HER2/neu in older age groups [18]. Additionally, our study revealed a HER2/neu overexpression in grade III tumors, consistent with the findings of Al-Temimi et al. of 100% membranous HER2/neu positivity in grade III tumors compared to grade I [19]. Our study demonstrated a statistically significant correlation between HER2/neu staining and lymph node metastasis, with six cases exhibiting HER2/neu overexpression in metastatic lymph nodes. This concurs with the results reported by Vani et al., where eight of the 30 cases with lymph node metastasis showed HER2/neu overexpression [20].

Furthermore, statistical significance was observed concerning the side of the tumor (left > right), stage of the tumor, and lymphovascular invasion, with p-values of 0.32, 0.0001, 0.008, and 0.47, respectively. Pyo et al. also noted a statistically significant relationship between HER2/neu overexpression and nodal metastasis, while no statistical significance was observed for HER2/neu overexpression with tumor depth [21].

The evolving landscape of CRC demographics, with an increasing incidence in individuals below 50 years, was underscored. Distinct clinical presentations in younger patients, emphasizing the importance of tailored diagnostic approaches, were highlighted. A flexible sigmoidoscopy is suggested for patients below 40 years with a history of rectal bleeding and no risk factors, whereas a full colonoscopy is recommended for patients over 40 years with additional risk factors [22].

HER2/neu emerged as an extensively studied therapeutic target for CRC, with variable outcomes reported in past decade-long studies. A phase II study on trastuzumab plus irinotecan in HER2/neu overexpressing tumors revealed a positive response in a subset of patients, indicating potential efficacy. However, the challenges in these studies, such as low accuracy rates and the influence of concurrent chemotherapy, were acknowledged. Comparatively, HER2-targeted therapy demonstrated promising outcomes in clinical trials when juxtaposed with treatment options for metastatic colon carcinoma, including immunotherapy and BRAF-directed therapy [23,24]. The incidence of HER2/neu amplification was found to be comparable to MSI-high tumors (5%) rather than BRAF mutations (10%).

The ongoing phase II HERACLES study explored the combination of oral lapatinib and trastuzumab in patients with HER2/neu overexpression, providing insights into potential therapeutic strategies [25]. Additionally, the MOUNTAINEER study, a phase II trial of tucatinib, an oral selective inhibitor of HER2/neu receptor, exhibited positive preliminary findings with a notable objective response rate (ORR) of 52.2% and a median response time of 10.4 months, further underscoring the therapeutic potential of HER2-targeted approaches in metastatic carcinoma. Our detailed analysis illuminates the nuanced relationship between HER2/neu status and clinicopathological features in CRC, offering valuable insights for future research and potential therapeutic strategies [26].

Strengths of the study

Careful Methodology

The study employed a meticulous methodology, including rigorous inclusion and exclusion criteria, standardized tissue processing, and validated IHC protocols. This ensured consistency in data collection and analysis.

Systematic Data Collection

Comprehensive clinical, histopathological, and molecular data were collected systematically, allowing for a detailed assessment of HER2/neu expression and its correlation with tumor characteristics.

Regional Significance

This study is one of the few to examine HER2/neu expression in CRC in the Indian subcontinent, offering fresh insights into the prevalence and clinical relevance of HER2/neu in this population. Given the regional differences in CRC biology, these findings contribute valuable data that can inform local diagnostic and therapeutic strategies.

Limitations of the study

The study's limitations include a smaller sample size, which poses a significant constraint, and the lack of FISH analysis, which hinders a comprehensive understanding of HER2/neu amplification in CRC.

Limited Sample Size

While the findings are significant, the relatively small cohort (40 patients) limits the generalizability of the results. A larger sample size would provide more robust statistical power and allow for a better understanding of HER2/neu expression in diverse CRC subtypes.

Challenges in IHC Evaluation

The lack of standardized HER2/neu scoring criteria for CRC introduces potential inconsistencies in IHC evaluation. The absence of a standardized protocol for assessing HER2/neu in CRC introduces variability across global studies, limiting the generalizability of the findings. This limitation underscores the need for CRC-specific guidelines.

Absence of Longitudinal Data

The cross-sectional nature of the study precludes the assessment of HER2/neu’s impact on long-term clinical outcomes, such as progression-free survival, overall survival, and response to HER2-targeted therapies.

Lack of Comparison With Other Biomarkers

HER2/neu expression was not compared with other relevant CRC biomarkers (e.g., KRAS, BRAF, and microsatellite instability), which could have provided a more comprehensive understanding of its role within the molecular landscape of CRC.

Absence of FISH Analysis

The lack of FISH analysis limits a comprehensive understanding of HER2/neu amplification in CRC. FISH is considered the gold standard for confirming HER2/neu gene amplification, especially in cases with equivocal (2+) IHC results, and its inclusion could have provided more robust and clinically relevant findings.

Recommendations

Future research should focus on large sample sizes, greater geographic diversity, and the incorporation of treatment regimens, along with long-term follow-up histories. Including longitudinal outcome data and additional biomarkers will provide a more holistic analysis. Establishing a standardized protocol for HER2/neu assessment in CRC is essential to ensure consistency across studies, enabling more robust comparisons and conclusive interpretations.

Moreover, incorporating gene amplification studies, particularly FISH analysis for cases with equivocal HER2/neu expression, would offer a deeper understanding of HER2/neu behavior in CRCs.

## Conclusions

The study findings emphasize that CRACs exhibit HER2/neu overexpression, supporting the use of IHC as an effective method for determining the frequency of this tumor marker. Histological grade and pathological stage of colorectal cancers are key factors influencing the prevalence of HER2/neu expression. These parameters serve as valuable predictive tools, contributing to the prognostication of CRC patients. For cases with equivocal or mixed results, the use of FISH analysis is recommended to confirm HER2/neu overexpression definitively.

Patients with high-grade, advanced-stage CRC, accompanied by lymph node metastasis and lymphovascular invasion, and who demonstrate HER2/neu overexpression are potential candidates for monoclonal antibody-based targeted therapy. This targeted approach holds promise for reducing the global mortality rate among CRC patients.
